# Valorization of the Hydrolate Byproduct from the Industrial Extraction of Purple *Alium sativum* Essential Oil as a Source of Nematicidal Products

**DOI:** 10.3390/life12060905

**Published:** 2022-06-17

**Authors:** Alberto Galisteo, Azucena González-Coloma, Purificación Castillo, María Fe Andrés

**Affiliations:** 1 Instituto de Ciencias Agrarias, Consejo Superior de Investigaciones Científicas (CSIC), 28006 Madrid, Spain; albertogapre@ica.csic.es (A.G.); azu@ica.csic.es (A.G.-C.); 2 Coopaman S.C.L., Las Pedroñeras, 16660 Cuenca, Spain; culviman@coopaman.com

**Keywords:** essential oil, garlic, hydrosol, nematicidal effects

## Abstract

The hydrolate byproduct resulting from the industrial essential oil extraction of Spanish purple garlic has been studied against the root-knot nematode *Meloidogyne javanica* by in vitro and in vivo bioassays. The essential oil, the hydrolate and its organic fraction caused high mortality of juveniles, suppressed egg hatch, and reduced nematode infection and reproduction on tomato plants. The nematicidal compounds of garlic oil, diallyl disulfide and diallyl trisulfide, were the major components of the hydrolate organic fraction. These findings have important implications for the development of new nematode control products based on garlic hydrolate compounds and highlight the recovery of waste from essential oils extraction, promoting a circular economy.

## 1. Introduction

Root-knot nematodes (*Meloidogyne* spp.) are major pathogens of crop vegetables, impacting both the quantity and quality of marketable yields. Among the more than 100 root-knot species described, *M. incognita* and *M. javanica* are the most damaging species, and are distributed worldwide, have a wide range of host plants and reproduce by parthenogenesis [1], and they cause losses estimated at tens of billions of euros year^−1^ [2]. *M. javanica* is often the dominant root-knot species, especially in areas of protected agriculture [3]. For decades, the control of these plant pathogens has been mainly conducted by non-fumigant and fumigant chemical nematicides [4]. Most of these nematicidal products are evidently groundwater contaminants, persistent in the soil, and produce serious adverse effects on the environment and human health. This has resulted in either restrictions in use or eventual withdrawal from the pesticides market [5,6], hence amplifying the need to screen less toxic and environmentally friendly substitutes. The global nematicides market is expected to continue growing and, with an increasing demand for synthetic chemical-free organic foods, botanical nematicides are taking the lead as replacements. Consequently, in recent years, there have been efforts towards identification of the active secondary metabolites from plants [7].

Essential oils (EOs) are obtained from the distillation process of aromatic plants, while the hydrolates or hydrosols (the decoction water in the case of hydrodistillation or the condensation water in the case of steam-distillation), are considered by-products or even wastes, often being discarded [8,9]. In agriculture, the pesticide effects of hydrolates have been extensively described and, specifically, the nematicidal potential of some hydrolates has been reported [9]. 

Garlic (*Allium sativum*), native to temperate western Asia, is one of the most important bulb crops in the world for its numerous culinary and medicinal uses, with an annual production of more than 30 million tons [10]. In Spain, some 36,000 hectares of garlic are cultivated with an annual production of 212,160 metric tons. The main garlic production area in Spain is La Mancha, with more than 17,000 hectares dedicated to this crop. The purple garlic variety (Ajo Morado de Las Pedroñeras) is an autochthonous ecotype with external tunics with a slightly tinted color, strong smell, and a spicy and stimulating taste due to its high content of organosulfides, especially allicin. Commercial purple garlic is presented in loose heads of a diameter of 45–41 mm for Extra or I categories, respectively. Therefore, the rest of the annual garlic production that does not meet the size requirements is a residue that can be processed to obtain a wide variety of products with culinary and pharmacological applications [11]. Garlic oil is produced commercially by steam distillation and is widely used in alimentary and pharmaceutical industries [12] and has a wide range of biological effects as an antioxidant [13], antibacterial [13], acaricide [14,15] and insecticide [16,17]. Specifically, nematicidal activity of this oil has been described against the pine wood nematode, *Bursaphelenchus xylophilus* [18] and the root-knot nematode *M. incognita* on cucumber [19] and tomato plants [20,21].

The extraction of garlic EO generates an aqueous residue, the hydrolate. Garlic hydrolates have demonstrated strong antibacterial activity against *Bacillus subtilis* and *Salmonella enteritidis* [22], however little is known regarding their nematicidal activity.

We hypothesized that garlic hydrolate byproduct, obtained from the steam distilla-tion of residual Spanish purple garlic bulbs, has a great nematicidal potential. Therefore, to test the above-mentioned hypothesis, we (i) studied the nematicidal effects of garlic EO, hydrolate and hydrolate organic fraction against *M. javanica* in vitro and in tomato plants. (ii) characterized their chemical composition (iii) discussed the garlic hydrolate valorization as a source of nematicidal products for root-knot nematode control.

## 2. Materials and Methods

### 2.1. Plant Material

The purple garlic plant is cultivated by Coopaman SA in Las Pedroñeras, Cuenca, Spain. At harvest, the undersize garlic bulbs (<36 mm) are considered garlic waste and were used for the preparation of the extracts.

### 2.2. Extraction and Fractionation

Undersize garlic bulbs were extracted by steam distillation in an industrial plant equipped with 1080 L. Garlic EO was separated from the aqueous phase, hydrolate, with a yield of 0.2% (*w*/*w*).

Garlic hydrolate was absorbed with activated carbon (2 L hydrolate/80 g of carbon) and stirred for 30 min at room temperature. The resulting activated carbon was separated from the liquid by filtration on a Büchner with a cellulose filter and dried at 45 °C for 24 h. The solid part was extracted with AcOEt in a Soxhlet for 12 h. The solvent was evaporated under reduced pressure to give 8.0 g of extract (hydrolate OF) with a yield of 0.032% (*w*/*v*).

### 2.3. Chemical Characterization: GC-MS Analysis

The volatile compounds of the essential oils and hydrolate extracts were analyzed by gas chromatography coupled to mass spectrometry (GC-MS) using a GC-2010 (Shimadzu, Kioto, Japan) equipment coupled to a GCMS-QP2010 (Shimadzu, Kioto, Japan) mass detector, equipped with a Simple Quadrupole analyzer, an automatic injector (AOC-20i) (Shimadzu, Kioto, Japan) and a (95%) dimethyl- (5%) diphenyl polysiloxane capillary column (30 µm × 0.25 mm ID and 0.25 µm phase thickness) ( Teknokroma TRB-5, Barcelona, Spain). The samples (in DCM) were detected by electronic impact at 70 e with Helium as a carrier gas. The working conditions were as follows: Split mode injection (1 µL injected), division ratio (20:1), injector temperature 300 °C, transfer line temperature 250 °C and ionization source temperature 220 °C. The initial temperature was 70 °C, heating up to 290 °C at 6 °C/min plus 20 min leaving at 290 °C. Mass spectra and retention time are used to identify compounds by comparison with the Wiley and NIST17 databases (Wiley 275 Mass Spectra Database, 2001, Palmer, Massachusetts, USA; NIST Mass Spectra Database, 2017, Gaithersburg, Maryland, USA).

### 2.4. Nematicidal Activity

The nematode population (*Melodogyne javanica*) is maintained on tomato (*Solanum lycopersicum* ‘Marmande’) pots in a grow chamber (25 ± 1 °C, 70% relative humidity). 

#### 2.4.1. In Vitro Effects on Juveniles 

Hand-picked egg masses of *M. javanica* from tomato roots incubated in a water suspension at 25 °C for 24 h were used to obtain second-stage juveniles (J2). Bioassays were carried out in 96-well plates (BD Falcon, San Jose, CA, USA) and treatments were replicated four times as described by Andrés et al. [23].

Filtered (25 µm) nematode juveniles (500) were included in 500 µL of hydrolate. Aliquots (100 µL) of the nematode suspension (approximately 100 J2) and controls (water) were placed in each well [24]. The extracts (OF hydrolate and EO and at 20 mg/mL) were dissolved in a 5% DMSO-Tween solution in water (0.5% Tween 20 in DMSO) and added (5 µL) to 95 µL of water containing 90–150 nematodes to give a final concentration of 1 mg/mL [25]. The control wells contained water/DMSO/Tween 20. The positive control was thymol (LC_50_ = 0.143 mg/mL). 

The plates were covered and maintained in the dark at 25 °C and the dead J2 counted (at 24, 48 and 72 h under a binocular microscope). The nematicidal activity results are presented as percent dead J2s corrected according to Schneider-Orelli’s formula [26]. Six serial concentrations (1–0.031 mg/mL) of each treatment were tested to obtain an effective lethal concentration (LC_50_ and LC_90_) by Probit analysis (STATGRAPHICS Centurion XVI, version 16.1.02, The Plains, Virginia).

#### 2.4.2. In Vitro Effect on Egg Hatching

Egg masses (three) were placed in each well of a 96-well plate containing OF hydrolate and EO solutions at LC_90_ concentrations (0.017 mg/mL and 0.015 mg/mL). The control wells contained water/DMSO/Tween 20. The bioassays were carried out with four replicates for each treatment. The plates were covered and maintained in the dark at 25 °C for 5 days after which the hatched J2s were counted, and the treatments were replaced with sterilized distilled water. The hatched J2s from egg masses were monitored weekly for 1 month until egg hatching was finished in the control [25]. Results values were transformed by Log10 (x + 1), analyzed by ANOVA, and means separated by LSD at *p* < 0.05. Relative hatch inhibition rates (compared with the controls) were calculated.

#### 2.4.3. Effect on Infection and Reproduction of *M. javanica* Population in Tomato Plants

The hydrolate OF and EO extracts were evaluated at two concentrations (0.25 and 0.125 mg/mL) in 1% ethanol. These treatments (100 mL) were applied to a pot containing 1000 g of the moistened substrate (sterile sandy/loam soil mixture) at the time of nematode inoculation (2000 *M. javanica* eggs) and incubated for 5 days in a growth chamber (25 ± 2 °C, 60% RH). After this period, 4-week-old tomato var. Marmande seedlings were transplanted, maintained for 60 days in a growth chamber (25 ± 2 °C, 60% RH, 16 h photoperiod) and fertilized with 50 mL of a 0.3% solution of 20-20-20 (N-P-K) every 10 days. There were five pots (replicate) for each treatment and this experiment was carried out in duplicate. At harvest, the whole root system from each pot was collected. Roots were washed free of soil, examined for counting the number of egg masses, and processed according to Hussey and Barker 1973 [27] to extract nematode eggs. Relative suppression rate of the hydrolate OF and EO extracts on eggs masses and number of eggs per plant was calculated. The infection frequency (IF: number of egg masses per plant divided by the number of eggs inoculated per pot) and the multiplication rate (MR: number of eggs per plant divided by the egg inoculum) were determined. Data from each treatment were transformed by Log10 (x + 1), analyzed separately by ANOVA and mean values were compared by LSD at *p* < 0.05 to determine significant differences in nematode population reproductive traits associated with treatment effects.

## 3. Results 

### 3.1. In Vitro Nematicidal Activity and Chemical Profiles of Garlic Hydrolate and Essential Oil

The in vitro nematicidal effects of purple garlic EO and hydrolate are shown in Table 1 and Table 2. The EO was very effective, with LC_50_ and LC_90_ values of 0.012 and 0.017 mg/mL, respectively (Table 1 and Table S1). The hydrolate induced maximum J2 mortality (100%) of *M. javanica* J2 after 72, 48 and 24 h exposure and continued being effective up to a dilution of 13% (LC_90_). Similarly, the hydrolate organic fraction (OF) induced strong lethal effects on J2 at the maximum dose (1 mg/mL), at all times evaluated with LC_50_ and LC_90_ values, similar to the EO (0.011 and 0.015 mg/mL) (Table 1 and Table S1).

The EO and hydrolate OF tested at LC_90_ concentrations strongly inhibited *M. javanica* egg hatching, with a final suppression rate ranging between 84.8 and 96.6%. After 5 days of incubation, the EO had a potent egg hatching inhibition effect (92.8%) that increased significantly over time once the egg masses were placed in water. Similarly, the hydrolate OF significantly suppressed egg hatching (88.3%) after 5 days of incubation and this effect was maintained (inhibition rate >80%) after the egg masses were immersed in water. 

The composition of the garlic essential oil (EO) and hydrolate organic fraction (OF) and are shown in Table 3 and Table S2. Diallyl trisulfide (DATS), diallyl trisulfide (DATS), and methylallyl trisulfide were the major components of the EO.

**Figure 1:**
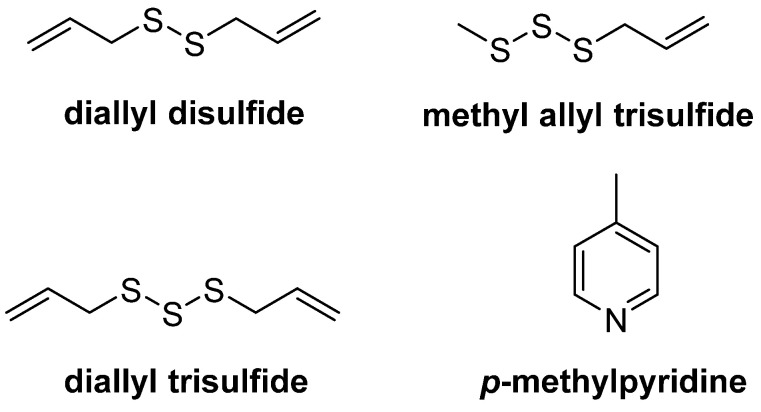
Major compounds in the organic fraction of hydrolate garlic.

A total of eight compounds (% abundance > 1) were identified in the hydrolate OF. The most abundant ones were diallyl disulfide (DADS), diallyl trisulfide (DATS), *p*-methylpyridine and methyl allyl trisulfide (Figure 1). 

### 3.2. Effects of Garlic Hydrolate and Essential Oil on Reproduction of Meloidogyne Javanica

Garlic essential oil (EO) and hydrolate OF-treated soils significantly reduced the reproductive traits of the *M. javanica* population (EMs, eggs/plant) and the IF and MR index, with respect to the untreated control in pot experiments (Table 4). The tomato plant growth was not influenced by these treatments (data not shown). The garlic EO at 0.025 mg/mL showed the highest effect, causing strong reductions (>90%) of nematode egg masses, egg production, and IF and MR, followed by the hydrolate OF at the same concentration and EO at 0.0125 mg/mL, with similar effects also causing strong suppression (>70%) of eggs masses and number of eggs per plant. Finally, the treatment with hydrolate OF at 0.015 mg/mL was less effective but demonstrated a significant activity on the nematode population parameters, resulting in relative suppression rates >50. 

## 4. Discussion

The garlic EO, the hydrolate and the hydrolate OF were very effective on *M. javanica* J2, indicating that the active components of the garlic hydrolate were present in the OF. Furthermore, the EO and hydrolate strongly inhibited *M. javanica* egg hatching. These results confirm the strong nematicidal activity of the EO and the hydrolate OF studied since the egg hatching inhibition effect indicates the ability of an extract/compound to penetrate the gelatinous mass matrix and affect the nematode eggs [10,27].

The nematicidal activity of garlic EO has been previously demonstrated in vitro against *B. xylophilus* [18] and to *M. incognita* in cucumber [19] and tomato plants [20,21]. However, this is the first report on the nematicidal effects of garlic hydrolate. There are previous reports on the strong in vitro nematicidal effects of garlic EO against *M. incognita*, with effective concentrations (CL_50_ and CL_90_ 0.033 and 0.076 mg/mL) more than two times higher than the ones reported here and lower capacity to suppress egg hatching [21]. These differences in garlic oil activity may be due to factors, such as the target *Meloidogyne* species (*M. javanica* vs. *M. incognita*), the garlic cultivar [28,29] and the distillation technique (industrial steam distillation vs. hydrodistillation) [30,31].

The in vitro effects of garlic hydrolate found here are greater than other highly active hydrolates such as *Thymus vulgaris* and *T. zygis* with CL_50_ and CL_90_ values ranging between 0.202–0.203 and 0.277–0.269 mg/mL, respectively [23].

The EO composition was dominated by allyl polysulfides, as previously reported for garlic essential oils from Spain [30]. Similarly, the most abundant components of the hydrolate OF were diallyl disulfide (DADS), diallyl trisulfide (DATS), *p*-methylpyridine and methyl allyl trisulfide. Hydrolates are normally composed of water-soluble oxygenated compounds also present in the EO [7,10,28,32]. The nematicidal activity of DADS and DATS has been demonstrated against the pine wood nematode, *B. xylophilus* [18], *M. javanica* [33], and *M. incognita* [21]; therefore, the nematicidal effects of garlic EO and hydrolate reported here can be explained by their content in active DADS (27.44%) and DATS (16.82%).

The in vivo results on tomato–nematode interaction corroborate the strong nematicidal effects shown by the EO and hydrolate of Spanish purple garlic against J2 and egg hatching of *M. javanica*. In vivo nematicidal effects of garlic EO against *M. incognita* on cucumber [19] and tomato plants [20,21] have been reported. Furthermore, similarly to our results, garlic oil obtained by hydrodistillation reduced the number of galls and eggs of *M. incognita* in tomato plants (at 0.2 mL/L of substrate) [21]. However, this is the first report on the in vivo nematicidal effects of garlic hydrolate.

The present work highlights the nematicidal potential demonstrated by garlic hydrolate, a byproduct obtained from the extraction of EO by industrial steam distillation. In recent years, a significant amount of research on the detection of nematicide products in hydrolates has been carried out. However, most studies tested aqueous extracts obtained by laboratory hydrodistillation (Clevenger apparatus) [34,35,36] without further processing, making these extracts difficult to standardize and replicate. The extraction of the organic fraction of the hydrolate allows quantifying the nematicidal activity as well as to standardize and characterize (chemical marker, chemical fingerprint) different batches of this active byproduct [9].

## 5. Conclusions

This study demonstrated, for the first time, the nematicidal activity against *M. javanica* of garlic hydrolate, a byproduct obtained from the extraction of EO by industrial steam distillation from Spanish garlic. Hydrolate treatments caused high mortality of juveniles, suppressed egg hatch, and reduced nematode infection and reproduction on tomato plants. Likewise, the powerful in vitro and in vivo nematicidal effects of garlic EO have been confirmed. The nematicidal compounds of garlic oil, DADS and DATS, were detected in the organic fraction of the hydrolate, also characterized as the major components. Garlic EO is one of the most valuable oils for its numerous applications in the food and pharmaceutical industries. Its production by industrial steam distillation generates a valuable hydrolate byproduct. These findings have important implications for the development of new nematode control products based on active compounds from garlic hydrolate and highlight the valorization of wastes from EO extraction, promoting the circular economy.

## Figures and Tables

**Table 1:** Nematicidal effects of hydrolate (HD), hydrolate organic fraction (HD-OF) and essential oil (EO) from *Allium sativum* on second–stage juvenile (J2) of *Meloidogyne javanica*.

Treatments	J2 Mortality (%) ^a^				
	24 h ^b^	48 h	72 h	LC_50_ (95% CL) ^c^	LC_90_ (95% CL)
**HD**	100	100	100	7.79 (7.23–7.97) ^d^	13.27 (12.63–14.03)
**HD-OF**	100	100	100	0.011 (0.010–0.011) ^e^	0.015 (0.0150–0.016)
**EO**	100	100	100	0.012 (0.011–0.013)	0.017(0.016–0.017)

^a^ Values (%) are means of four replicates. ^b^ Incubation time of treatments: undiluted hydrolate and hydrolate OF and essential oil at 1 mg/mL. ^c^ At least six concentrations/dilutions were used, at 72 h, to obtain LC_50_ and LC_90_. CL denotes confidence limit. ^d^ Values are % v HD/v water. ^e^ Values are mg OF/mL HD.

**Table 2:** Effects of hydrolate organic fraction and essential oil from *Allium sativum* on *Meloidogyne javanica* egg hatching with time.

Treatments	Hatched Juveniles and Relative Hatch Suppression Rate (%) * with Time **								
	0		7		14		21	28	
**Control** (H_2_O)	190.25 ± 6.52 ***^,a^		368.75 ± 11.93 ^a^		571.5 ± 22.09 ^a^		114.5 ± 12.82 ^a^	12 ± 2.73 ^a^	
**EO** (0.015 mg/mL)	13.58 ± 3.92 ^b^	92.8 *	33.74 ± 3.63 ^b^	90.8	2.28 ± 0.62 ^b^	99.6	1 ± 0.40 ^b^	0 ^b^	100
**HD-OF** (0.017 mg/mL)	22.25 ± 3.68 ^c^	88.3	50.15 ± 10.53 ^c^	86.4	86.86 ± 9.36 ^c^	84.8	21.31 ± 4.55 ^c^	2 ± 0.70 ^b^	83.5

* Time 0: after 5 days of immersion in test solutions; time 7 and subsequent times: number of days of immersion in water after time 0. ** Values are mean ± standard error of hatched juveniles from three egg masses/four replicates. Values within the same column followed by different lower-case letters are significantly different according to Least Significant Difference (LSD) test (*p* < 0.05). *** Each value represents the hatch inhibition rate in the respective treatment corrected according to the control.

**Table 3:** Chemical composition of the hydrolate organic fraction and essential oil from *Allium sativum* analyzed by GC-MS.

Compound	Rt	% Abundance EO	% Abundance Hydrolate OF
*p*-methyl pyridine	2.99		18.22
methyl 2-propenyl disulfide	3.57	5.48	4.70
diallyl disulfide	6.09	31.31	27.44
(E)-1-allyl-2-(prop-1-en-1-yl)disulfane	6.51		2.15
methyl allyl trisulfide	7.29	12.25	10.63
2-vinyl-4H-1,3-dithiine	8.89	1.31	1.01
2-methyl-3-(methylthio) furan	9.05		2.53
diallyl trisulfide	10.81	26.58	16.82

**Table 4:** In vivo effects of hydrolate and essential oil on reproductive traits of *Meloidogyne javanica* in tomato plants, 60 days post-inoculation, with 2000 eggs per plant, maintained in a growth chamber.

Treatments	Egg Masses/Plant *	RS ** %	Eggs/Plant ×100 *	RS *** %	IF ****	MR *****
**Control**	197.1 ± 20.3 ^a^		1411 ± 120 ^a^		0.09855	70.55
**EO** [0.125 mg/mL]	47.4 ± 6.2 ^c^	76	385 ± 86 ^c^	73	0.0237	19.25
**EO** [0.250 mg/mL]	11.6 ± 1.5 ^d^	94	53 ± 10 ^d^	96	0.0058	2.65
**HD-OF** [0.125 mg/mL]	75 ± 5 ^b^	62	588 ± 67 ^b^	58	0.0375	29.4
**HD-OF** [0.250 mg/mL]	48 ± 5.1 ^c^	76	396 ± 70 ^c^	71	0.024	19.8

* Values are mean ± standard error of ten replicated plants. Values within the same column followed by different lower-case letters are significantly different according to Least Significant Difference (LSD) test (*p* < 0.05). ** Relative suppression on eggs masses per plant. *** Relative suppression on number of eggs per plant. **** Infection Frequency: egg masses per plant/egg inoculum. ***** Multiplication Rate: eggs per plant/egg inoculum.

## Supplementary Materials

The following supporting information can be downloaded at: https://www.mdpi.com/article/10.3390/life12060905/s1, Table S1: Estimated lethal concentrations (LC_50_ and LC_90_) of the essential oil, hydrolate and hydrolate organic fraction against *Meloidogyne javanica* juveniles after 72 h.; Table S2: Complete dates of chemical analysis by GC-MS and library identification of the hydrolate organic fraction and essential oil from *Allium sativum.* Refs [37,38] are mentioned in the Supplementary file.
