# Supplementary material for: Valorization of the Hydrolate Byproduct from the Industrial Extraction of Purple Alium sativum Essential Oil as a Source of Nematicidal Products
Source: Life (Basel). 2022 Jun 17;12(6):905. doi: 10.3390/life12060905 (PMC9228356; doi:10.3390/life12060905)
Supplement: Supplementary file 1 [file life-12-00905-s001.zip › life-1737547-supplementary.pdf]

Valorization of the Hydrolate Byproduct From the Industrial Extraction of Purple Alium sativum Essential Oil as a Source of Nematicidal Products

Alberto Galisteo [1], Azucena González-Coloma [1], Purificación Castillo [2] and María Fe Andrés [1,*]

[1] Institute of Agriculture Sciences, CSIC, 28006, Madrid, Spain; albertogapre@ica.csic.es (A.G.); azu@ica.csic.es (A.G.C.)
[2] COOPAMAN S.C.L. culviman@coopaman.com (P.C.)
* Correspondence: mafay@ica.csic.es (M.F.A.)

List of contents

- Table S1
- Table S2

Table S1: Estimated lethal concentrations (LC_{50} and LC_{90}) of the essential oil, hydrolate and hydrolate organic fraction against *Meloidogyne javanica* juveniles after 72 h.

**Treatments**	**Intercept**	**Standard Error**	**Slope**	**LC_{50} (95% Confidence interval)**	**LC_{90} (95% Confidence interval)**
**Essential oil**	2.81	0.067	240.46	0.012 (0.011-0.0130)*	0.017(0.016-0.0175)
**Hydrolate OF**	2.81	0.062	264.88	0.0106 (0.0102-0.0110)*	0.0154 (0.0150-0.0158)
**Hydrolate**	1.71	0.084	0.22	7.79 (7.23-7.97)**	13.27 (12.63-14.03)

* Values are mg / mL. ** Values are % v HD / v water

Table S2: Complete dates of chemical analysis by GC-MS and library identification of the hydrolate organic fraction and essential oil from *Allium sativum*.

Compound	Rt	RI	% Abundance EO	% Abundance Hydrolate OF	m/z	Identification
p-methyl pyridine	2.99	874		18.22	93/66/92/65/67/39/94/63/40/51	NIST
methyl 2-propenyl disulfide	3.57	916	5.48	4.70	120/41/39/45/73/78/122/80/63/121	NIST*
diallyl disulfide	6.09	1077	31.31	27.44	41/81/113/39/79/85/45/105/73/112	NIST*
(E)-1-allyl-2-(prop-1-en-1-yl)disulfane	6.51	1100		2.15	81/41/73/45/146/104/39/61/105/71	NIST
methyl allyl trisulfide	7.29	1137	12.25	10.63	87/73/41/45/39/47/88/78/110/63	NIST*
2-vinyl-4H-1,3-dithiine	8.89	1212	1.31	1.01	72/71/111/144/97/45/79/39/103/73	NIST*
2-methyl-3-(methylthio) furan	9.05	1219		2.53	128/99/65/110/113/67/53/85/66/39	NIST
diallyl trisulfide	10.81	1298	26.58	16.82	113/73/41/45/39/79/114/71/72/47	NIST*

* These compounds were compared with the m/z spectrum of the compounds of the EO in literature [1,2].

References

1. Yu, T.H.; Wu, C.M.; Liou, Y.C. Volatile compounds from garlic. *J. Agric. Food Chem.* **1989**, *37*, 725–730.
2. Hu, G.; Cai, K.; Li, Y.; Hui, T.; Wang, Z.; Chen, C.; Xu, B.; Zhang, D. Significant inhibition of garlic essential oilon benzo[a]pyrene formation in charcoal-grilled pork sausages relates to sulfide compounds. *Food Res. Int.* **2021**, *141*, 110127, doi:10.1016/j.foodres.2021.110127.
